# Variations in Respiratory Excretion of Carbon Dioxide Can Be Used to Calculate Pulmonary Blood Flow

**DOI:** 10.14740/jocmr1979w

**Published:** 2014-11-19

**Authors:** David A. Preiss, Takafumi Azami, Richard D. Urman

**Affiliations:** a Department of Anesthesia, Critical Care, and Pain Medicine, Harvard Medical School/Massachusetts General Hospital, Boston, MA, USA; b Department of Pathophysiology and Anesthesia, Nagoya City University School of Nursing, Nagoya City, Japan; c Department of Anesthesia, Perioperative and Pain Medicine, Harvard Medical School/Brigham and Women’s Hospital, Boston, MA, USA

**Keywords:** Pulmonary blood flow, Cardiac output, Carbon dioxide elimination, End-tidal carbon dioxide

## Abstract

**Background:**

A non-invasive means of measuring pulmonary blood flow (PBF) would have numerous benefits in medicine. Traditionally, respiratory-based methods require breathing maneuvers, partial rebreathing, or foreign gas mixing because exhaled CO_2_ volume on a per-breath basis does not accurately represent alveolar exchange of CO_2_. We hypothesized that if the dilutional effect of the functional residual capacity was accounted for, the relationship between the calculated volume of CO_2_ removed per breath and the alveolar partial pressure of CO_2_ would be reversely linear.

**Methods:**

A computer model was developed that uses variable tidal breathing to calculate CO_2_ removal per breath at the level of the alveoli. We iterated estimates for functional residual capacity to create the best linear fit of alveolar CO_2_ pressure and CO_2_ elimination for 10 minutes of breathing and incorporated the volume of CO_2_ elimination into the Fick equation to calculate PBF.

**Results:**

The relationship between alveolar pressure of CO_2_ and CO_2_ elimination produced an R^2^ = 0.83. The optimal functional residual capacity differed from the “actual” capacity by 0.25 L (8.3%). The repeatability coefficient leveled at 0.09 at 10 breaths and the difference between the PBF calculated by the model and the preset blood flow was 0.62 ± 0.53 L/minute.

**Conclusions:**

With variations in tidal breathing, a linear relationship exists between alveolar CO_2_ pressure and CO_2_ elimination. Existing technology may be used to calculate CO_2_ elimination during quiet breathing and might therefore be used to accurately calculate PBF in humans with healthy lungs.

## Introduction

A clinician’s ability to determine a patient’s cardiac output is critical in the assessment of cardiopulmonary health. Clinically, this measurement can be required intermittently during surgery, as well as post-operatively. Cardiac output may also be measured in research settings, but doing so with invasive methods requires expensive critical care equipment, trained personnel, a sterile environment and readily available resuscitative equipment.

Cardiac output is arguably one of the most important parameters reflecting cardiovascular health and yet its measurement is currently limited to patients with Swan-Ganz catheters in the operating room, critical and intensive care units. To date, no non-invasive method has proven itself comparable to thermodilution in accuracy, repeatability, and in physiological soundness [1, 2]. Nevertheless, the thermodilution method is plagued with limitations in clinical practice, the most important ones being associated with its invasiveness [3].

Methods for measuring cardiac output non-invasively can be classified as respiratory-based or non-respiratory-based. The latter, which includes Doppler (external, transtracheal and transesophageal), bioimpedance, and pulse contour analysis, has not yet been accepted into clinical practice because of either theoretical or practical limitations [4]. Additionally, there are some respiratory-based methods that utilize foreign gas breathing, including argon and acetylene, but these methods require a source of external gas, are impractical in many settings, and will not be dealt with in detail here [5].

The respiratory-based methods are the oldest, the most physiologically sound, and traditionally the most accepted method of cardiac output measurement. Their principles and assumptions are well understood as are their limitations, the most important of which is that they more accurately measure pulmonary blood flow (PBF) rather than cardiac output. Originally described for oxygen, Fick equation, developed in the late 1800s, is based on the measurement of elements of the mass balance across the lungs [6]. The following is the equivalent equation describing the movement of carbon dioxide (CO_2_):

Eq. 1: PBF = VCO_2_/(CvCO_2_ - CaCO_2_)

where PBF is the pulmonary blood flow, VCO_2_ is the metabolic CO_2_ production, and CvCO_2_ and CaCO_2_ are the concentrations of CO_2_ in the blood entering (mixed venous, v) and leaving (arterial, a) the lungs respectively. To keep the method purely non-invasive, alveolar pressure is conventionally used as a surrogate for content. The relationship between PaCO_2_ and CaCO_2_ is usually assumed to be linear in the physiologic range, and through previous *in-vitro*, CO_2_ content of oxygenated blood can be estimated using the following equation [7, 8]:

Eq. 2: Content = 4 × Pressure + 260

Generally, PaCO_2_ is approximated from alveolar CO_2_ pressure (P_A_CO_2_), which in turn is approximated from end-tidal CO_2_ pressure (P_ET_CO_2_). Mixed venous pressure of CO_2_ (PvCO_2_) however, is difficult to obtain non-invasively, and has classically posed the biggest challenge to measurement of PBF. Respiratory maneuvers such as breathholding and rapid equilibration with external reservoirs have been employed to estimate PvCO_2_, but these have the disadvantage of requiring an external supply of CO_2_, and may pose a challenge to patients with respiratory compromise [9]. Single-breath methods have been presented, but none has proven itself repeatable and accurate.

In 1980, a novel method of calculating PBF without the need for measuring CvCO_2_ was described by Gedeon et al [9]. That method demonstrated that if a subject’s alveolar ventilation (V_A_) were acutely and transiently reduced, a step change in P_A_CO_2_ and VCO_2_ would result (once steady state is reached, after a few breaths). At this point, assuming PBF and PvCO_2_ had not changed in this short time (< 30 - 45 s), two Fick equations could be applied to the model:

Eq. 3a: PBF = VCO_2_/(S × PvCO_2_ - S × P_A_CO_2_)

Eq. 3b: PBF = VCO_2_’/(S × PvCO_2_ - S × P_A_CO_2_’)

where VCO_2_’ and P_A_CO_2_’ are the exhaled CO_2_ volumes and alveolar CO_2_ pressures after a new steady state has occurred, respectively, and S is a conversion constant to content. Since PvCO_2_ and PBF remain unchanged with this maneuver, these equations together yield a single equation:

Eq. 4: PBF = (VCO_2_ - VCO_2_’)/(S × P_A_CO_2_’ - S × P_A_CO_2_)

This way, a small, temporary change in V_A_ can be used to calculate PBF without the need for invasive monitors. This method has been proven reliable in intubated patients by multiple studies [10-12].

Creative as this method is; however, there are several limitations to it. First, the time to reach the new steady state is a function of the subject’s V_A_ and functional residual capacity (FRC). On average, this time is approximately 30 - 45 s, or about five breaths, which means that only two data points are used to calculate PBF before recirculation of arterial blood occurs, limiting the method’s accuracy. Second, because V_A_ is required to be transiently reduced, and because the normal variability of breathing creates noise in the measurement signal, this method is limited only to intubated patients. Finally, P_A_CO_2_ must be allowed to re-equilibrate to steady state levels before a subsequent test can be performed, which may require an additional 60 - 120 s.

## Materials and Methods

### Flux of CO_2_ at the alveoli

The method proposed here redefines the term VCO_2_ as described in Fick’s equation. According to conventional understanding, VCO_2_ is defined as the excretion of CO_2_ past the lips, measured with a pneumotachometer and CO_2_ monitor. However, exhaled CO_2_ on a breath-by-breath basis seldom reflects actual metabolic CO_2_ production or pulmonary capillary excretion into the lungs [13]. Instead, an average over several breaths is required for this estimation, as the FRC acts as a reservoir for CO_2_. For example, a large breath that expunges a large volume of CO_2_ past the lips would incorrectly reflect a large “production” of CO_2_. The Fick equation, therefore, would be more physiologically accurate if the term VCO_2_ represented the continuous flux of CO_2_ from the blood into the alveoli (VCO_2A_), rather than the discrete, tidal excretion of CO_2_ past the lips (VCO_2M_). Averaged over many breaths, VCO_2M_ will accurately reflect VCO_2A_.

### Relationship between VCO_2A_ and P_A_CO_2_

**Figure 1:**
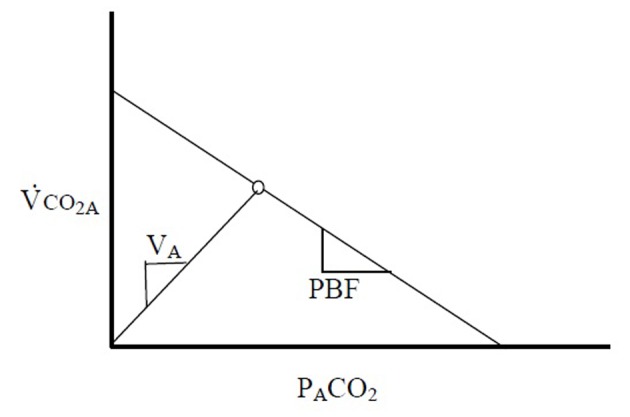
The relationship between VCO_2A_ and P_A_CO_2_ is linear. The slope of this line is proportional to the pulmonary blood flow. An intersecting line can be drawn from the origin following the relationship V_A_ = VCO_2_ × P_A_CO_2_/713. The steady state point (A) for P_A_CO_2_ and VCO_2A_ is produced by the balance of CO_2_ diffusion into the alveoli and the flow of blood into the pulmonary capillaries.

After redefining VCO_2_ in this manner, it becomes clear that there exists a linear relationship between the flux of CO_2_ out of the blood (VCO_2A_) and P_A_CO_2_ (Fig. 1).

At steady state VCO_2A_ and P_A_CO_2_ produce a single point on this line. The slope of this line, PBF, is proportional to the PBF. A second theoretical line exists on this diagram, connecting the origin with the steady state point. This second line is described by the basic physiologic equation:

Eq. 5: VCO_2A_ = V_A_ × P_A_CO_2_/713

where V_A_ is the alveolar ventilation - the slope of the line. It becomes clear that VCO_2A_ - not VCO_2M_ - must be applied when describing these relationships, as washout of the FRC can confound the changes that relate these variables.

**Figure 2:**
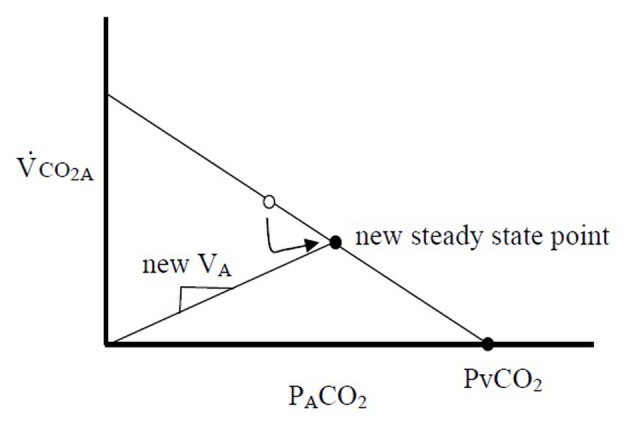
Gedeon’s method of reducing V_A_ for several breaths acutely increases P_A_CO_2_ and reduces VCO_2A_ creating a new steady state point on the same line. Point A and point B can be used together to calculate the slope of the line, which is the pulmonary blood flow.

Gedeon’s method aims to use two steady state VCO_2M_ points to determine the slope of the PBF line: one attained from the subject breathing at rest (where VCO_2M_ only equals VCO_2A_ if averaged over many breaths), and the second after a small change in V_A_ (measured after 4 - 5 breaths, when, after a transient period, VCO_2M_ is assumed to be equal to VCO_2A_). In an extreme case, if the breath were held for 30 s and a new steady state were reached, P_A_CO_2_ would be equal to PvCO_2_, or the x-intercept (Fig. 2). This method can only measure two points because after 4 - 5 breaths of breathing at a second V_A_, recirculation would alter PvCO_2_. However, if VCO_2A_ can be measured with each breath, one data point along this line could be produced with each exhalation, and the slope, PBF could be calculated with sequential breaths. Variations in VCO_2A_ are needed to explore this line, and the slope of the second line, V_A_, would vary with each breath due to normal variations in tidal volume and respiratory frequency. Since VCO_2A_ likely fluctuates little during quiet breathing (as opposed to VCO_2M_), variability of breathing, which was once the system “noise”, therefore becomes important for measurement of PBF.

Once this line is observed over multiple breaths, PBF can be calculated by extrapolating to the x-intercept, PvCO_2_, and calculating PBF using equation 1.

### Method for calculating VCO_2A_

The method described above requires calculation of the VCO_2A_, the flux of CO_2_ across the alveolar membrane. Calculation for VCO_2A_ has been described thoroughly elsewhere [13]. Briefly, a simple mass balance dictates that the flux of CO_2_ into the alveoli from the blood is related to the flux of CO_2_ at the mouth and the change of CO_2_ stores in the lung:

Eq. 6: VCO_2A_ = VCO_2M_ - ΔVCO_2S_

where VCO_2A_ is the alveolar flux of CO_2_, VCO_2M_ is the flux of CO_2_ at the mouth, and ΔVCO_2S_ is the change in lung stores of CO_2_. VCO_2M_ is easily measured using a metabolic cart, which integrates exhaled CO_2_ concentration and expiratory flow.

The change in lung stores of CO_2_ from breath 1 to breath 2, ΔVCO_2S_, can be described as the sum of two terms describing the change in CO_2_ concentration at a constant V_A_, and the change in V_A_ at a constant CO_2_ concentration:

Eq. 7: ΔVCO_2S_ = V_A_(P_A_CO_2_’ - P_A_CO_2_)/713 + P_A_CO_2_’ΔV_A_

where ΔVCO_2S_ is the change in alveolar stores of CO_2_, V_A,1_ is the alveolar volume at the end of breath 1, P_A_CO_2_ and P_A_CO_2_’ are the fractions of CO_2_ in the alveoli at the end of breaths 1 and 2, respectively and ΔV_A_ is the change in alveolar volume from breath 1 to breath 2.

### Computer simulation

For this study, a computer simulation of tidal breathing was designed to test the theory under ideal conditions using Microsoft Excel 2003 (Redmond, Washington, USA). Incremental calculations of lung CO_2_ volume were made for each 0.001 min. Tidal breathing was simulated using variable inhaled and exhaled tidal volumes (±30%), allowing lung volume to return to a different FRC with each breath. Complete alveolar mixing was assumed, inspiratory and expiratory times were equal, inhalation and exhalation flows were linear, and alveolar dead-space and shunt were assumed to be minimal. P_A_CO_2_ was recorded once per breath (the final P_A_CO_2_ value at the end of exhalation) and VCO_2M_ was calculated at the mouth during exhalation. The model was run over a period of 10 min (100 breaths), and P_A_CO_2_, VCO_2M_, Vt-in and Vt-out were recorded with each breath. V_A_ for sequential breaths was calculated as V_A_’ = V_A_ + Vt-in - Vt-out, where Vt-in and Vt-out are inhaled tidal volume and exhaled tidal volume, respectively. Detailed parameters are outlined in Table 1.

**Table 1:** The Parameters Used in the Mathematical Model

Parameter	Error	Value
PvCO_2_ (mm Hg)	None	50
Pulmonary blood flow (L/min)	None	6
Tidal volume (mL)	±30%	500
Respiratory frequency (/min)	None	12
FRC (L)	None	3
PBF-preset (L/min)	None	6

### P_A_CO_2_-ave versus P_ET_CO_2_

As stated above, the diffusion of CO_2_ across the alveolar membrane should form a linear relationship with the pressure of CO_2_ in the alveoli. Therefore, measurements of P_ET_CO_2_, a single sample taken at the end of exhalation, would be inappropriate to use in equation 3 since it reflects an end-expiration value rather than an average (P_A_CO_2_-ave). In the computer model, an average P_A_CO_2_ is easy to calculate, but this is not the case clinically, when P_ET_CO_2_ samples are the only measurements readily available non-invasively. To estimate P_A_CO_2_-ave for a single breath using only non-invasive data, we back-calculated to the P_A_CO_2_ that might exist at peak inhalation and averaged this with P_ET_CO_2_. The P_A_CO_2_ at peak inhalation might be estimated as:

Eq. 8: (Volume of CO_2_ in Lung at End-Exhalation - Volume of CO_2_ Diffused during Exhalation + Exhaled Volume of CO_2_) × 713/Peak Alveolar Volume

Averaging this value with P_ET_CO_2_ may produce a reasonable estimate of P_A_CO_2_-ave which can be applied in equation 1.

### Calculation of VCO_2A_

VCO_2S_ was calculated using equation 6 using sequential breaths, ΔV_A_ was assumed to be measurable without the need for nitrogen monitors (Vt-in and Vt-out were measurable), and VA was initially assumed to be 3 L. VCO_2A_ was then calculated using equation 5. Using 10 min of breathing data, when PBF and PvCO_2_ were assumed to be in steady state, a plot of VCO_2A_ versus P_A_CO_2_ was created and R^2^ was calculated for the line of best fit. FRC was then iterated in increments of 0.25 L from 2 L to 4 L to achieve the most optimal linear relationship between VCO_2A_ and P_A_CO_2_.

### Real-world adjustments

To simulate real-world measurements, error was introduced into each measurement (P_A_CO_2_, Vt-in, Vt-out, VCO_2M_, Table 1), consistent with manufacturer specifications [14] and calculation of PBF was repeated.

### PBF comparisons

PBF was calculated from equation 3 using two techniques: first, using known VCO_2A_ measurements directly from the model (PBF-preset), calculated using actual diffusion of CO_2_ across the alveolar membrane, and second, using VCO_2M_ to calculate VCO_2A_ using equation 5 (PBF-calc), which represents the strategy that might be used practically in subjects. In the either case, PBF would be calculated using equation 1 where PvCO_2_ was attained by extrapolating to the x-intercept of a plot relating VCO_2A_ and P_A_CO_2_-ave. We determined the appropriate number of breaths required for accurate measurement of PBF-calc, by increasing the number of breaths used to calculate the average PBF-calc until the repeatability was similar to that of thermodilution [15].

PBF-calc was assessed and evaluated using Bland-Altman analysis where the acceptable error was taken from prior established theory [16].

## Results

**Figure 3:**
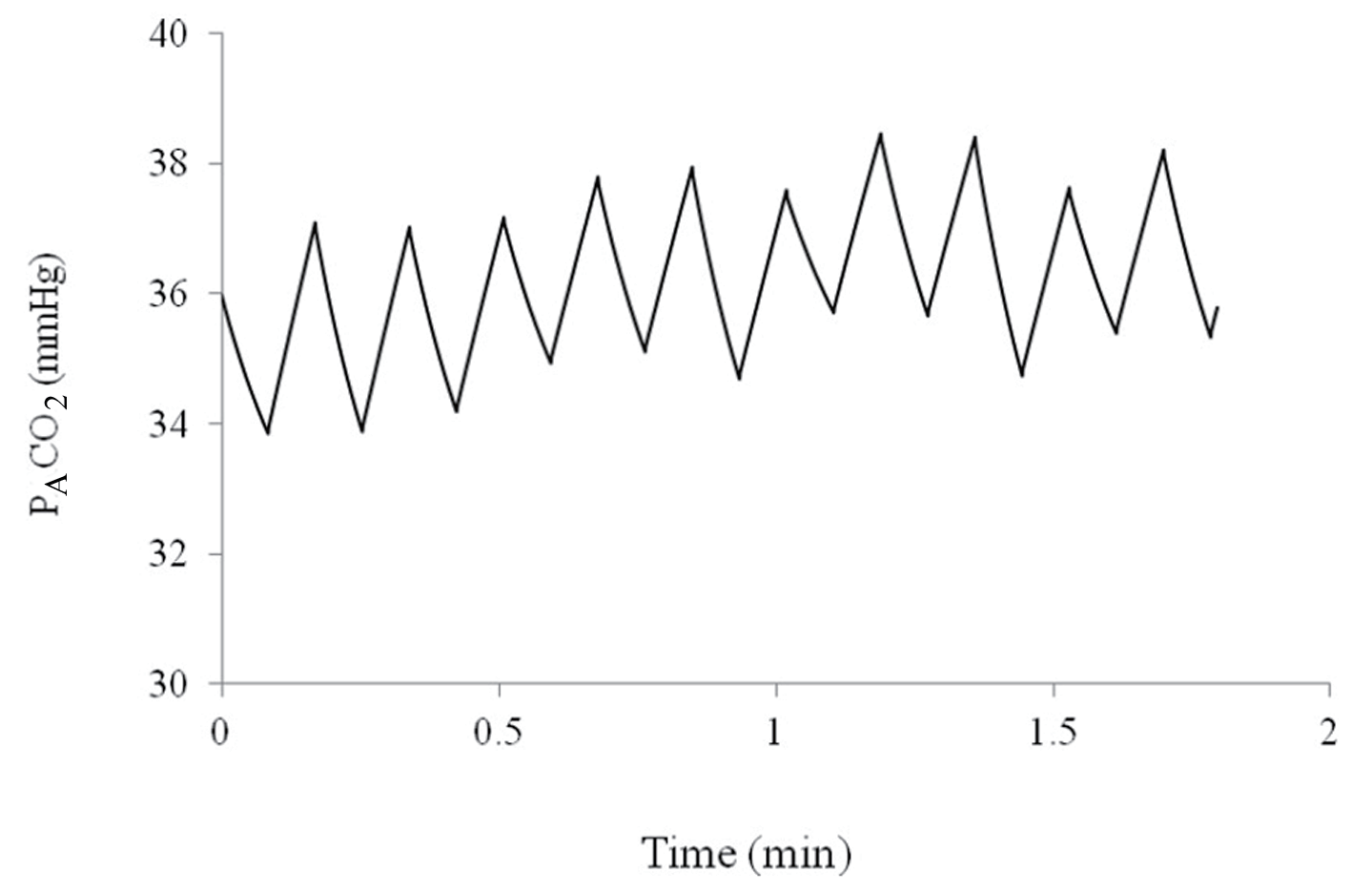
Alveolar partial pressure of CO_2_ over a period of 2 min as predicted by the computer model.

The model for tidal ventilation produces reasonable values for P_A_CO_2_ over 10 min of breathing. Sample oscillations in P_A_CO_2_ and lung volume can be seen in Figure 3.

**Figure 4:**
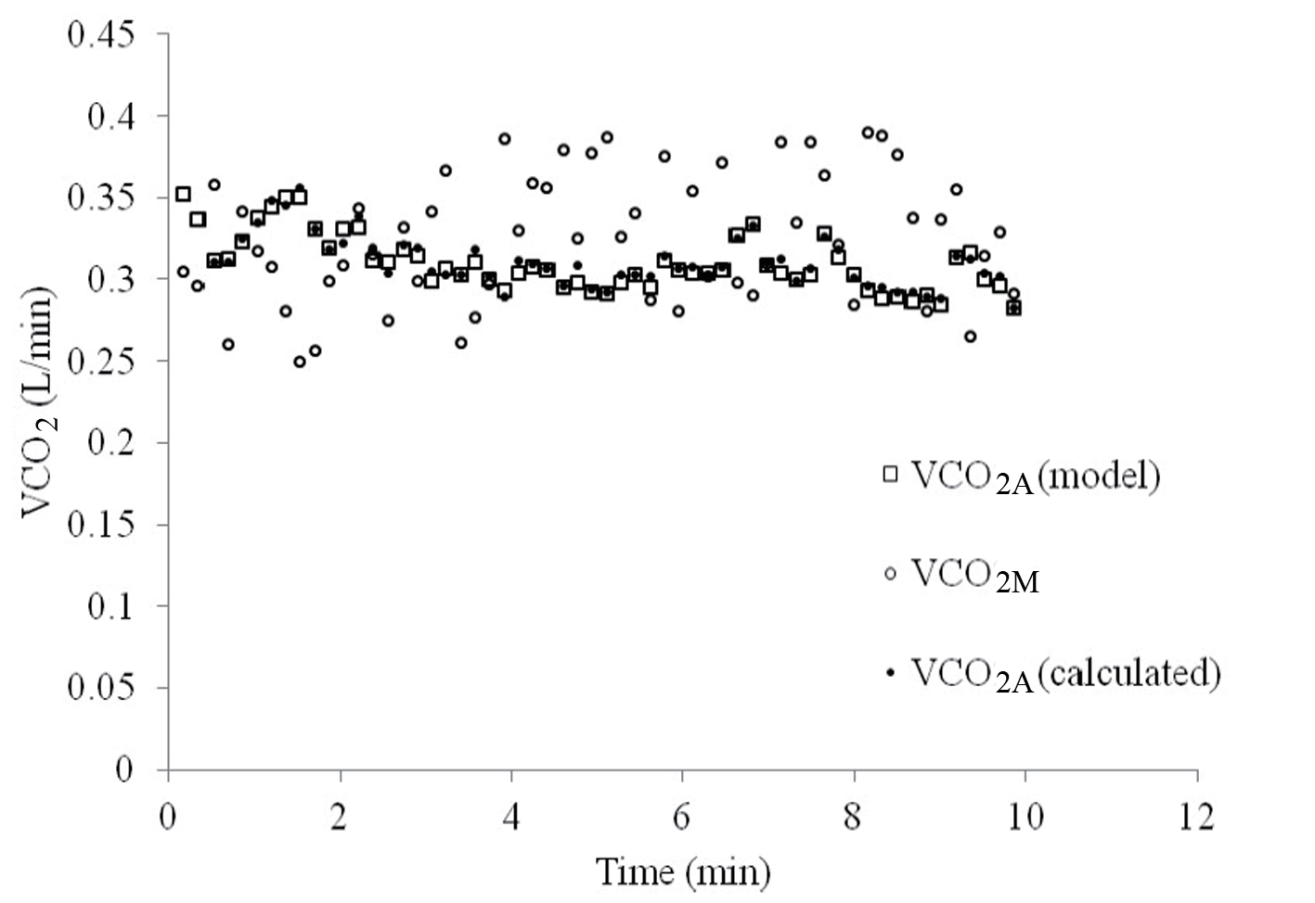
VCO_2 _over time as measured at the mouth (VCO_2M_, open circles), as pre-set by the model (VCO_2A_-model, open squares) and as calculated using the proposed method (VCO_2A_-calculated, closed circles).

**Figure 5:**
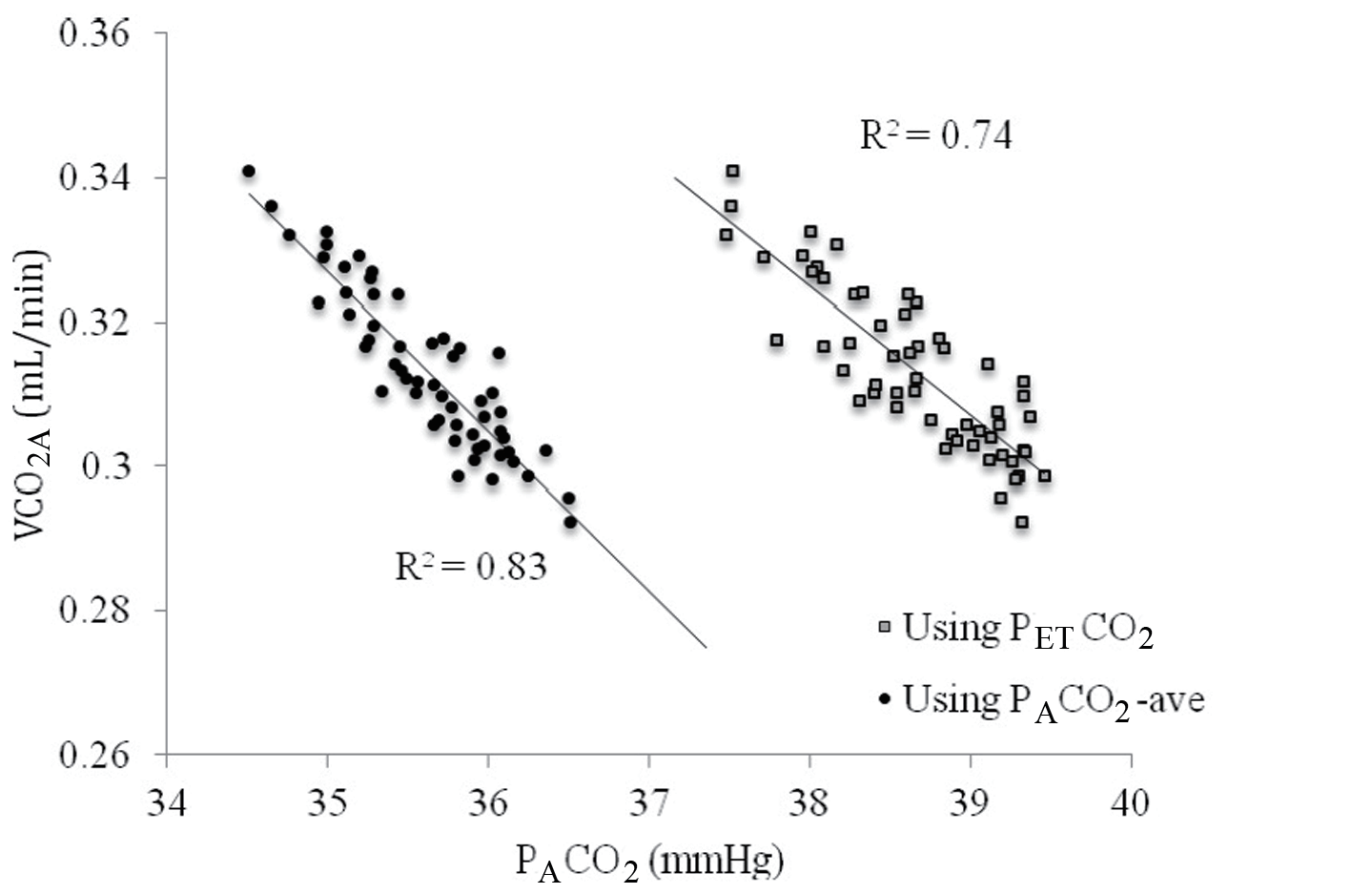
The relationship between VCO_2A_ and P_A_CO_2_ using end-tidal PCO_2_ (P_ET_CO_2_, gray squares) versus time averaged CO_2_ across the breath (P_A_CO_2_-ave, closed circles).

VCO_2A_ tended to vary less over the course of 10 min of breathing than VCO_2M_, as can be seen in Figure 4. When VCO_2A_ was plotted against P_A_CO_2_-ave a linear relationship was revealed which was stronger than with P_ET_CO_2_ (R = 0.83 versus 0.74, Fig. 5).

**Figure 6:**
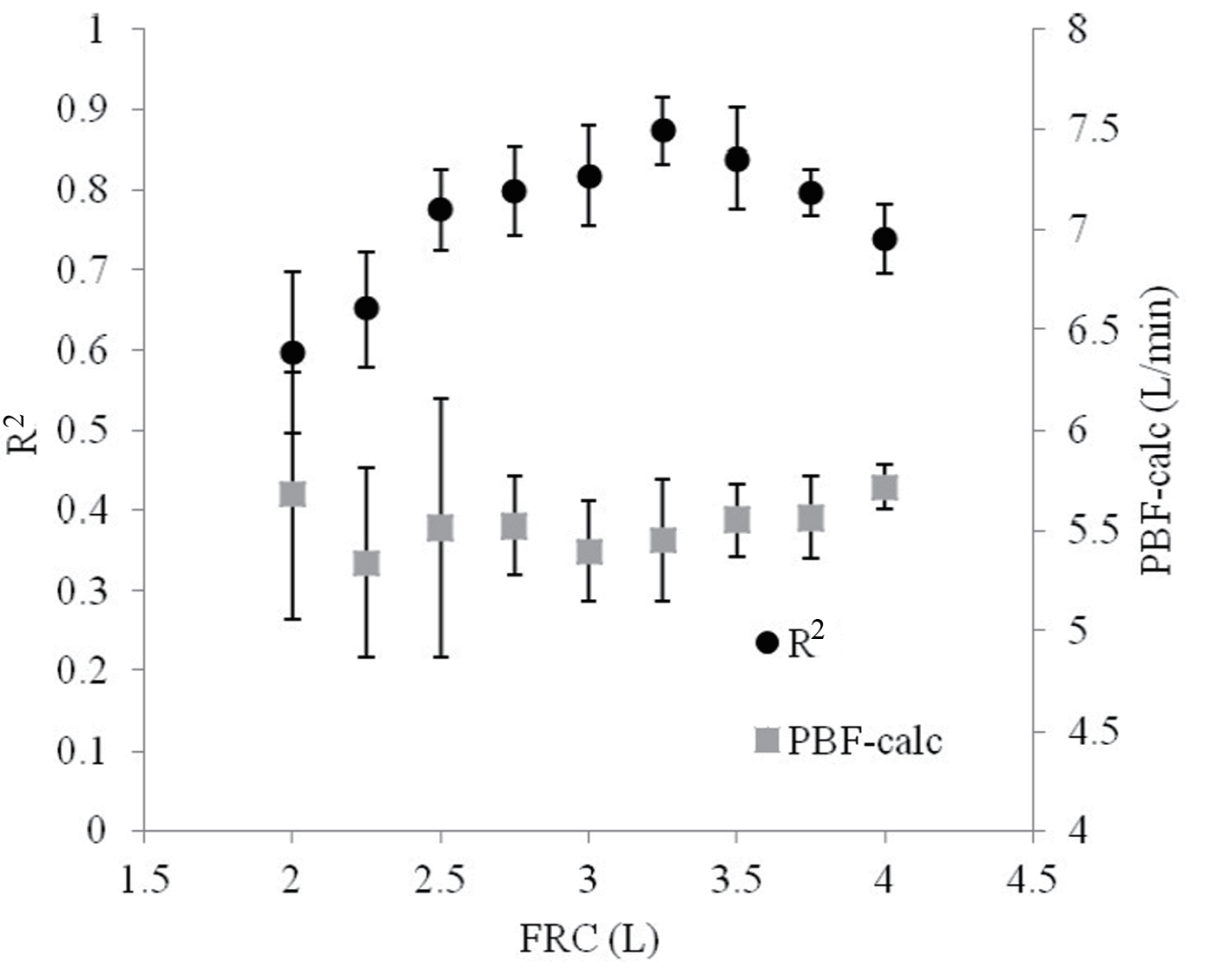
The R^2^ statistical parameter relating VCO_2A_ to P_A_CO_2_ varies depending on the original estimate of FRC. As the initial guess of FRC is increased, R^2^ reaches a peak, optimum point and then falls.

Iterating FRC demonstrated that small deviations from the model value produced poorer relationships between VCO_2A_ and P_A_CO_2_ (Fig. 6). However, the optimal FRC achieved after iterating FRC was 3.25 L, which was 0.25 L (8.3%) greater than the actual FRC used in the model. Non-optimal FRCs did not significantly reduce the accuracy of the calculated PBF, but did increase its variability.

When P_A_CO_2_-ave was used instead of P_ET_CO_2_, the relationship was improved (R^2^ = 0.83 versus 0.74). PBF-calc was calculated using 10 breaths at a time (see repeatability below) by extrapolating the line in Figure 3 to the x-intercept (PvCO_2_) and applied into equation 1. Using this method, the difference between PBF-preset and PBF-calc was 0.62 ± 0.53 L/min.

**Figure 7:**
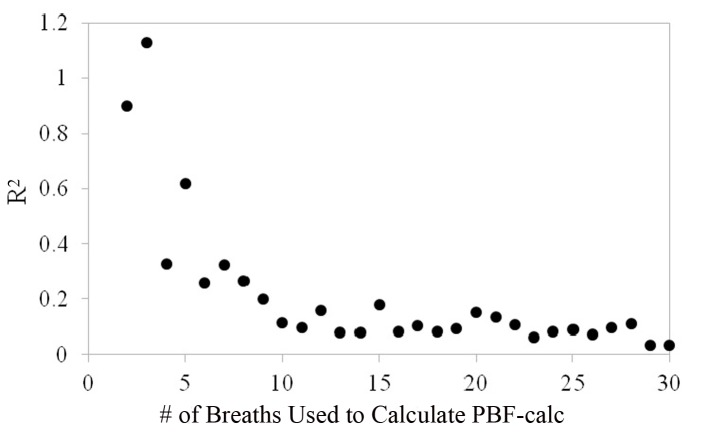
As few as two breaths can be used to calculate PBF but its reliability is increased as more breaths are incorporated into the calculation. Precision reaches a plateau at about 8 - 10 breaths.

Figure 7 shows that the repeatability coefficient fell from 1.13 to 0.09 L/min as the number of breaths used to calculate PBF-calc increased from 2 to 30. The repeatability coefficient decreased and leveled at 0.09 when approximately 10 breaths were used to average calculation for PBF-calc. The repeatability coefficient for thermodilution is likely between 0.4 and 0.6 L/min [4, 15]), which is the equivalent of using 5 - 6 breaths to average PBF-calc measurements in the proposed method.

## Discussion

The most common method for cardiac output measurement involves indicator dilution, normally dyes and temperature. A dye, or cold saline, is typically injected via an invasive catheter into the pulmonary artery and the temporal profile of the concentration of the dye or the temperature of the blood is measured downstream. The profile of the indicator change over time is used to calculate the cardiac output. Over the last three decades, there has been a steady improvement in the technology required to manufacture the catheters and thermistors, and to analyze the indicator curves and calculate the cardiac output [17]. In addition, the expertise to place the catheters and look after catheterized patients has become widespread.

Nevertheless, there are at least three drawbacks necessarily associated with these methods. First, pulmonary artery catheters are inherently invasive and have associated complications including damage to the carotid artery, subclavian artery and lung, air emboli, pneumothorax, malignant arrhythmias and heart block, rupture of right atrium, right ventricle and/or pulmonary capillary, local infection and septicaemia, and more [3]. Second, they have many associated costs as a result of the requirement for critical care areas, equipment and personnel. Third, their accuracy is questionable, and can be less reliable and helpful as required for management of critically ill patients [18]. Despite these drawbacks, thermodilution methods continue to be widely used as less invasive alternatives are not sufficiently accurate, not sufficiently robust, or too cumbersome to perform in a large variety of clinical settings [19].

In this study, a new non-invasive method of measuring PBF is introduced based on principles that are already accepted in medicine. The Fick method for measuring PBF is well established and is considered one of the most accurate techniques available. Until recently, however, the Fick technique could only be performed using blood samples despite numerous attempts to measure PvCO_2_ non-invasively. The method of creating a step-change in V_A_ is the only validated non-invasive Fick method of cardiac output measurement without a special maneuver required by the patient, but can only be used on patients ventilated by a mechanical ventilator, uses only two breaths for measurement ETC. If a spontaneously breathing patient is made to rebreathe previously exhaled gas, the minute ventilation will tend to increase in order to increase the volume of air entering the lungs for gas exchange. The method presented here would require no maneuver on the part of the subject, and no foreign or compressed gases.

The method outlined here describes an original relationship between VCO_2_ and P_A_CO_2_. Its principles are grounded in basic physiology, and its application may extend beyond that of PBF measurement. Since P_A_CO_2_ relates directly to VCO_2_ given a specific set of conditions, other variables that may influence these parameters such as alveolar dead space, may be measureable as well.

There are several practical limitations of this method. First, this strategy for measuring PBF could not be achieved without a perfect, air-tight seal around the mouth, nose, or face. Any air lost would reduce the accuracy of Vt-in, Vt-out, or VCO_2M_. This may be inconvenient or impossible for some patients, depending on the presence of facial hair, anomalous anatomy, or trauma.

This model assumes that all exhaled gas had participated in exchange of CO_2_ with the blood. P_A_CO_2_ may vary depending on differences in ventilation-to-perfusion matching throughout the lung [2, 20]. If some exhaled gas originated from alveolar dead space, P_ET_CO_2_ would underestimate P_A_CO_2_ and as a result, PBF-calc would overestimate PBF. The significance of this was not quantified in this study, but theoretically, it is possible that iterations of alveolar dead space estimates can be coupled with the estimates of FRC to provide an optimal relationship between P_A_CO_2_ and VCO_2A_. Still, deviations of P_ET_CO_2_ from P_A_CO_2_ may also be due to incomplete breaths during exhalation and not alveolar deap space per se. This also impacts the calculation for P_A_CO_2_-ave, which will also be affected by all of the above.

In this study, 10 min of breathing were permitted to achieve initial FRC estimate. At this point, it is unclear why the ideal FRC produced from the iterative process differed from that used in the model. Nevertheless, its impact on PBF-calc was marginal. Furthermore, the purpose of this extended period of breathing was to demonstrate the principle rather than practicality. Follow-up studies in humans will be required to further explore this strategy.

The real-world error in measuring Vt, P_ET_CO_2_, VCO_2_ was based on products currently available for purchase. These will vary depending on the manufacturer and may improve in the future, making this method more practical.

The accuracy of this method for measuring PBF was similar to others that have been proposed [15]. The repeatability demonstrated that 10 breaths were needed for optimal accuracy, a time slightly greater than would be required for a complete test of thermodilution (about 30 s) depending on respiratory frequency. However, one advantage of the proposed method in this respect is that measurements for PBF are continuous, and no time is needed for “reset”, as may be needed to washout indicator from the pulmonary artery for thermodilution.

Finally, whereas the accuracy of other respiratory-based non-invasive methods may be diminished by respiratory fluctuations, the proposed method is enhanced by large changes in tidal volume and respiratory frequency. It may even be suggested that a subject ought to be encouraged to take deep breaths, or sigh to exaggerate the variability of quiet tidal breathing.

### Conclusion

The study we describe here is safe, theoretically sound, and demonstrates acceptable accuracy and repeatability. It represents a potential advancement towards measurement of important cardiopulmonary parameters that may be clinically important in the management of both outpatients and those in-hospital. Further studies in humans are required to quantify and evaluate its strengths and limitations.

## Source(s) of Support

Funding for this study was provided by internal department funds.
